# Real world outcomes of distributing Lucira Check-It® COVID self-tests in Ontario, Canada: the GetaKit COVID study

**DOI:** 10.1186/s12889-024-17783-9

**Published:** 2024-02-15

**Authors:** Lauren Orser, Janet E. Squires, Alexandra Musten, Nikki Ho, Jennifer Lindsay, Nitika Pant Pai, Patrick O’Byrne

**Affiliations:** 1 Faculty of Health Sciences, School of Nursing, University of Ottawa, 451 Smyth Rd, Ottawa, ON K1H 8M5 Canada; 2 Department of Medicine, School of Population and Global Health, McGill University, 5252 Boulevard de Maisonneuve W., Montréal, QC H4A 3S5 Canada

**Keywords:** GetaKit, Self-testing, Online testing, Rapid testing, COVID, Health services

## Abstract

**Background:**

In Ontario, Canada we developed and implemented an online screening algorithm for the distribution of HIV self-tests, known as GetaKit. During the COVID pandemic, we adapted the GetaKit algorithm to screen for COVID based on population and infection data and distributed COVID rt-LAMP self-tests (using the Lucira Check-It®) to eligible participants.

**Methods:**

GetaKit/COVID was a prospective observational study that occurred over a 7-month period from September 2021 to April 2022. All potential participants completed an online registration and risk assessment, including demographic information, COVID symptoms and risk factors, and vaccination status. Bivariate comparisons were performed for three outcomes: results reporting status, vaccination status, and COVID diagnosis status. Data were analysed using Chi-Square for categorial covariates and Independent Samples T-Test and Mann-Whitney U test for continuous covariates. Bivariate logistic regression models were applied to examine associations between the covariates and outcomes.

**Results:**

During the study period, we distributed 6469 COVID self-tests to 4160 eligible participants; 46% identified as Black, Indigenous or a Person of Colour (BIPOC). Nearly 70% of participants reported their COVID self-test results; 304 of which were positive. Overall, 91% also reported being vaccinated against COVID. Statistical analysis found living with five or fewer people, having tested for COVID previously, and being fully vaccinated were positive factors in results reporting. For COVID vaccination, people from large urban centers, who identified their ethnicity as white, and who reported previous COVID testing were more likely to be fully vaccinated. Finally, being identified as a contact of someone who had tested positive for COVID and the presence of COVID-related symptoms were found to be positive factors in diagnosis.

**Conclusions:**

While most participants who accessed this service were vaccinated against COVID and the majority of diagnoses were identified in participants who had symptoms of, or an exposure to, COVID, our program was able to appropriately link participants to recommended follow-up based on reported risks and results. These findings highlight the utility of online screening algorithms to provide health services, particularly for persons with historical barriers to healthcare access, such as BIPOC or lower-income groups.

## Introduction

Because case management – including self-isolation and contact tracing – of people diagnosed with SARS-COV-2 2019 (henceforth “COVID”) was one key intervention to limit onward transmission of this infection [1, 2], the COVID pandemic highlighted the need for expansive access to testing for such respiratory viral infections [2]. Indeed, without testing, diagnosis and subsequent case and contact management could not have occurred. While clinician-administered testing was the main method of diagnosis at the beginning of the COVID pandemic, self-testing increased during the latter part, not only due to infections caused by the Omicron variant overwhelming clinical and laboratory facilities [2, 3], but also because self-test devices enabled people to obtain results at home without the risk either of transmission to others or of infection acquisition while en route to, or waiting at, a testing location [2, 4]. Reducing the number of persons who sought in-person clinical testing was also a strategy to decrease healthcare costs and limit transmission to healthcare providers for all viral infections with COVID-like symptoms [4].

To evaluate the uptake, acceptability, and feasibility of self-testing, we developed a website (GetaKit.ca/COVID) through which persons could register and complete a risk self-assessment. The results of the self-assessment [5] dictated participants next steps, which included: (1) recommendations to seek in-person care, (2) the distribution of 1 or 2 COVID self-tests via mail to a participant’s home address, or (3) no indication for testing. For this study, we used the Lucira Check-It® real-time LAMP (rt-LAMP) self-test, with the goal of determining the outcomes of an online system for distributing these tests. While we have previously described the overall uptake of this project (O’Byrne et al. [5]), herein we report on the correlates of those who reported test results, those who reported positive test results, and those who reported being vaccinated against COVID. These results shed light on implications for online access to self-testing.

## Methods

GetaKit.ca/COVID was a prospective observational open cohort study and was an expansion of the pre-existing GetaKit.ca study [6], which, at the time we implemented the COVID self-test expansion, only distributed free HIV self-tests in Ontario, Canada using an HIV risk assessment algorithm [7]. This COVID arm of this study ran until we distributed the 6500 test kits allotted to us by Health Canada, which was from September 14, 2021 to December 19, 2021 via the website (GetaKit.ca/COVID), with targeted outreach from January 1, 2022 to April 19, 2022 through community-based agencies that provide services to and for persons of African, Caribbean, and Black ethnicities and to and for members of Indigenous communities.

To be eligible, participants had to be: 16 years of age or older, residing in Ontario, experiencing one or more COVID-specific symptoms and/or having COVID-related risk factors (including being identified as a contact of someone who tested positive for COVID), residing in an area or being a member of a population with elevated COVID prevalence (e.g., African, Caribbean, or Black, or Indigenous), in a household with 5 or more people, or having a chronic health condition. The rationale for focusing on these populations was that, at the time of this study, the members of these groups were experiencing the highest rates of COVID infection – with some having the greatest barriers to accessing healthcare (including COVID testing). Our goal, therefore, was not to engage in mass population-level screening, but rather, to distribute COVID self-tests precisely to persons with a higher pretest probability for COVID infection. Meanwhile, those who were under the age of 16, living outside of Ontario, reported no COVID related risk factors, or who reported severe COVID symptoms were ineligible for testing, and those with severe symptoms were advised to seek in-person assessment with a healthcare provider.

### Recruitment

We shared information about the GetaKit.ca/COVID study via social media (Twitter, Instagram, Facebook), via mainstream media (newspapers, radio, television), and by official government communications. Recruitment informed potential participants about the study and encouraged them to access the website to determine their eligibility for free COVID self-tests.

### Data collection

For this study, we created a new risk assessment questionnaire based on Government of Ontario Ministry of Health guidelines [8], which included questions about symptoms, COVID exposures, the use of personal protective equipment, and vaccination status. Participants also provided basic demographic information, including age, gender, income, living arrangements, and address. (See Table 1 for a list of data collection questions and variables.)

**Table 1:** List of Data Collection Questions and Variables

Category	Questions
Demographics	• Address • Age • Ethnicity • Income • Gender
Risk contact	• Living arrangement o Number of persons (if living with others) o If these people are immunocompromised (if living with others) • Personal health conditions
Risk assessment	• Prescence of symptoms o Severity of symptoms (if present) o Type of symptoms (if present) • Contact of COVID or someone with COVID-like symptoms o How notified (if contact) - Informed by person with COVID - Informed by COVID app - Informed by local health unit o When (if contact) o Use of PPE (if contact)
Vaccination	• Vaccine status • Interest in information about vaccines (if unvaccinated)
Testing preferences	• Ever tested before? o When (if tested) o Why never tested before (if not tested) • Preferred type of testing

Based on these risk assessment data [8], the GetaKit.ca/COVID algorithm stratified participants as no/low, medium, or high risk – and then immediately provided participants with recommendations for testing based on their answers. Participants were therefore not immediately eligible to obtain COVID self-tests; instead, they were invited to complete the risk assessment to see which services were best suited for them. Those who were no/low risk were ineligible for testing. Those deemed medium risk were offered 1 self-test. Those deemed high risk were offered 2 self-tests. Those with severe symptoms [8] were denied testing and instructed to contact emergency services; details about how to do so were provided by the system based on participants’ geo-location. Participants whose answers indicated the need for emergency services were also locked out of the GetaKit.ca/COVID system for 48 hours. All participants could reorder every 7 days.

Our rationale for distributing tests to participants in this way was to ensure that we tailored services based on participant need. Persons without risk factors did not need testing, whereas those with severe symptoms needed immediate in-person care. Both were therefore denied self-tests and given relevant instructions through GetaKit.ca/COVID. Persons with medium risk (per the GetaKit.ca/COVID algorithm), meanwhile, needed testing, but had only a moderate pretest probability of infection. They therefore only received 1 self-test. Those who were higher risk for infection (e.g., *bona fide* contacts or symptomatic) were given 2 self-tests so they could do one of the following: [1] if their first self-test was positive: give the second test to someone else they had been in contact with; [2] if their first self-test was negative: perform the second self-test at 7–10 days from possible COVID exposure or symptom onset to complete the test outside the COVID window period; or [3] if their first self-test was invalid: complete the second test immediately. Our intention was to distribute higher numbers of self-tests into networks where infections were present.

All participants received two automated messages by email asking them to report their results via GetaKit.ca/COVID. Members of the research team phoned all participants who did not report a self-test result to inquire if they would share this information. The options for reporting results were positive, negative, invalid, and prefer not to report. Participants were also required to report one of the foregoing test results if they attempted to re-order a self-test.

All data for this study were stored on a secured encrypted website that was housed in Canada. These data were extracted into an MS Excel CSV file for analysis.

### Test device

Our GetaKit.ca/COVID study used the Lucira Check-It® COVID self-test, which employed real-time LAMP (i.e., molecular amplification) technology in a single-use test to detect RNA of the N gene for SARS-COV-2 from self-collected nasal swabs [9]. The test could identify a positive result in 11 min but would cycle for up to 30 min to yield negative or invalid results [9]. The test was approved by Health Canada [10] for persons 14 years of age and older for both persons with and without symptoms. According to the device manufacturer, the Lucira Check-It® had a sensitivity of 92–100%, with a positive percent agreement of 97% when the cycle threshold was ≤ 37.5, and a specificity of 98–100% [9].

### Data analysis

Sample characteristics for categorical variables were presented as counts (percentages) and, for continuous variables, as means (standard deviation, SD). Bivariate comparisons between groups were performed for the three outcomes: reporting status (reported COVID results, did not report COVID results), vaccination status (fully vaccinated, not fully vaccinated), and COVID status (positive, negative), using the Chi-Square Statistic for categorial covariates and the Independent Samples T-test and Mann–Whitney U test for the continuous covariates. A two-tailed *p*-value < 0.05 was considered to represent statistical significance. Simple and multiple binary logistic regression models were then fitted to examine unadjusted and adjusted associations of demographic factors, housing factors, and COVID health factors on the three outcomes. Selection of covariates for entry into the regression models was by statistical selection following the bivariate analyses; all covariates with *p*-values of < 0.10 were selected for model entry. For the outcome *reporting status*, covariates entered in the model included: ethnicity, income, gender, age, live with (number of people), live with at risk persons, confirmed contact with COVID, tested for COVID before, and vaccination status. For the outcome *vaccination status*, covariates entered in the model included: population size, ethnicity, income, gender, live with (# of people), live with at risk persons, confirmed contact with COVID, experiencing COVID symptoms, tested for COVID before, preferred testing method, and reporting status. For the outcome *COVID status*, covariates entered in the model included: population size, ethnicity, age, confirmed contact with COVID, and experiencing COVID symptoms. For the final models, we report the adjusted odds ratios and 95% confidence intervals. The descriptive statistics, bivariate analyses and logistic regression analyses were performed using the Statistical Package for Social Sciences (SPSS) Version 28.0.

### Funding

This project was funded by the Ontario HIV Treatment Network, the Ministry of Health (Ontario), and Health Canada. Funders were not involved in data collection, analysis, or write-up, and had no influence on this paper. Health Canada purchased and imported all Lucira Check-It® tests, the Ministry of Health provided funding for study operations (staffing, mailing, website), and the Ontario HIV Treatment Network provided the base funding for the initial HIV study.

### Ethical approvals

The University of Ottawa Research Ethics Board approved this study (H-12-20-6450).

## Results

### Demographic characteristics

From September 14, 2021 to April 30, 2022, 7781 orders were placed for a COVID self-test through our study. In total, 53% (*n* = 4160) were deemed eligible by the GetaKit.ca/COVID algorithm; a total of 6469 COVID self-tests were distributed to these 4160 eligible orders. The mean age of participants who ordered these self-tests was 40.5 years (SD = 12.7). For these 4160 orders, 69% (*n* = 2879) had a result reported and 304 were positive, yielding a positivity rate of 4.7% for all distributed tests (*n* = 304/6469) and 7.3% for all unique orders (*n* = 304/4160). The majority (*n* = 3340) of these orders came from people who lived in large urban centres. For ethnicity, 53% (*n* = 2212) identified as white and 46% (*n* = 1905) as Black, Indigenous, or a Person of Colour (BIPOC). For gender identity, two-thirds (*n* = 2679) identified as cis-female, one-third (*n* = 1315) as cis-male, and 3% (*n* = 113) as trans or non-binary.

For preventative care, 91% (*n* = 3772) of participants reported being fully vaccinated against COVID (which at the time this study occurred was defined as at least 2 vaccine doses). Nearly two-thirds (*n* = 2712) of participants reported having completed prior testing for COVID.

Lastly, despite seeking COVID self-tests through our website GetaKit.ca/COVID, only 56% (*n* = 2310) of participants indicated that their preferred method of testing was by self-test, with the remaining 44% of participants preferring in-person testing from healthcare professionals. By preference for self-testing versus in-clinic testing, we did not identify any significant findings related to age (*p* = 0.451), gender (her vs. him vs. they, *p* = 0.079), ethnicity (white compared to BIPOC, *p* = 0.442), or income (less versus more than $75,000, *p* = 0.539).

### Reasons for obtaining COVID self-testing

Reasons for obtaining COVID self-testing through GetaKit.ca/COVID were divided into housing factors (living arrangements) and health factors (related to COVID). For housing, 88% of participants (*n* = 3639) reported living with others, of whom 65% (*n* = 2709) resided with 2–5 persons. Among those in multi-person households, half (*n* = 2060) reported living with ≥ 1 persons at-risk for COVID based on age, disability, and/or health condition. For health factors, one-quarter (*n* = 1075) of participants reported close contact with a person diagnosed with COVID and 16% (*n* = 654) reported COVID-like symptoms. Another 30% (*n* = 1236) reported having a health condition that might put them at risk of complications were a COVID infection to occur.

### Factors predicting results reporting

Statistical analyses identified some predictors between participants who did (*n* = 2880) or did not (*n* = 1280) report their COVID self-test results to GetaKit.ca/COVID. Those with an annual income <$25,000 were less likely to report their results compared to those who earned >$75,000. In addition, participants who identified as confirmed contacts of COVID had lower reporting rates compared to those who did not have specific concerns related to COVID. Reporting practices also decreased slightly with age. Participants who were more likely to report their results were those who had previously completed testing for COVID, who reported being fully vaccinated against COVID, and who reported living with 5 or fewer people (See Table 2).

**Table 2:** Results of Logistic Regression, with Binary Outcome Variable: Reporting Status

Covariates	Unadjusted		Adjusted	
	Odds Ratio (95% Confidence Interval)	*P*-Value	Odds Ratio (95% Confidence Interval)	*P*-Value
Ethnicity	1.748 (1.530, 1.997)	< 0.001	1.155 (0.902, 1.478)	0.253
Income				
<$25,000	0.509 (0.410, 0.631)	< 0.001	0.663 (0.473, 0.928	0.017
$25,000 - $49,999	0.649 (0.529, 0.798)	< 0.001	0.782 (0.571, 1.071)	0.126
$50,000 - $74,999	0.893 (0.723, 1.103)	0.294	1.079 (0.793, 1.469)	0.627
Gender				
Female	0.528 (0.286, 0.973)	0.041	0.839 (0.349, 2.015)	0.693
Male	0.566 (0.305, 1.050)	0.071	0.804 (0.331, 1.952)	0.630
Age	0.992 (0.987, 0.997)	< 0.001	0.989 (0.981, 0.998)	0.013
Live with (# People)				
1 person	1.916 (1.440, 2.549)	< 0.001	1.603 (1.007, 2.553)	0.047
2–5 people	1.706 (1.341, 2.169)	< 0.001	1.640 (1.151, 2.336)	0.006
Live with at Risk Persons				
Age	0.725 (0.555, 0.947)	0.018	0.782 (0.558, 1.094)	0.151
Health Condition	1.452 (1.165, 1.809)	< 0.001	1.103 (0.838, 1.453)	0.484
Disability	1.929 (1.067, 3.487)	0.030	1.331 (0.698, 2.538)	0.385
Confirmed Contact with Covid	0.848 (0.731, 0.983)	0.029	0.735 (0.564, 0.956)	0.022
Tested for Covid Before	1.811 (1.580, 2.076)	< 0.001	1.415 (1.109, 1.807)	0.005
Vaccination Status	2.203 (1.767, 2.745)	< 0.001	1.624 (1.058, 2.491)	0.027

Outcome (Reporting Status): reported Covid 19 results vs. did not report Covid-19 results

Reference Categories for Covariates: Ethnicity - reference category was BIPOC; Income – reference category was > $75,000; Gender – reference category was nonbinary; Live with (# People) - reference category was > 5 people; Live with at Risk Persons – reference category was combination of conditions; Confirmed Contact with Covid – reference category was no confirmed contact; Tested for Covid before – reference category was not tested before; Vaccination Status – reference category was not fully vaccinated

### Factors predicting COVID vaccination

Analyses were completed to examine predictors of COVID vaccination (*n* = 3772 fully vaccinated, *n* = 353 not fully vaccinated). Participants from large population centres were more likely to report being fully vaccinated against COVID compared to those from small population centres (1,000–29,999 people). Participants who reported their ethnicity as white and having previously reported their COVID testing results were also more likely to report full vaccination against COVID, compared to those who reported being BIPOC and who did not previously report their COVID testing results. Conversely, participants who reported their annual income as <$50,000 and living with at risk individuals based on age alone were less likely to be vaccinated against COVID compared to participants in high income brackets (annual income >$75,000) and those living with at risk individuals due to multiple conditions (See Table 3).

**Table 3:** Results of Logistic Regression, with Binary Outcome Variable: Vaccination Status

Covariates	Unadjusted		Adjusted	
	Odds Ratio (95% Confidence Interval)	*P*-Value	Odds Ratio (95% Confidence Interval)	*P*-Value
Population Size				
Large Urban	0.816 (0.567, 1.175)	0.275	1.968 (1.046, 3.702)	0.036
Medium	0.751 (0.453, 1.244)	0.266	1.135 (0.505, 2.551)	0.760
Ethnicity	2.874 (2.271, 3.637)	< 0.001	2.006 (1.238, 3.251)	0.005
Income				
<$25,000	0.212 (0.148, 0.304)	< 0.001	0.285 (0.158, 0.514)	< 0.001
$25,000 - $49,999	0.304 (0.211, 0.439)	< 0.001	0.492 (0.268, 0.904)	0.022
$50,000 - $74,999	0.429 (0.292, 0.631)	< 0.001	0.715 (0.375, 1.363)	0.308
Gender				
Female	0.169 (0.023, 1.222)	0.078	0.483 (0.062, 3.736)	0.485
Male	0.152 (0.021, 1.102)	0.062	0.507 (0.065, 3.970)	0.517
Live with (# People)				
1 person	2.782 (1.710, 4.527)	< 0.001	1.997 (0.855, 4.663)	0.110
2–5 people	1.512 (1.057, 2.161)	0.023	1.694 (0.972, 2.950)	0.063
Live with at Risk Persons				
Age	0.556 (0.360, 0.857)	0.008	0.468 (0.269, 0.814)	0.007
Health Condition	1.929 (1.245, 2.991)	0.003	1.258 (0.727, 2.177)	0.412
Disability	0.889 (0.366, 2.163)	0.796	0.694 (0.249, 1.928)	0.483
Confirmed Contact with Covid	2.315 (1.698, 3.157)	< 0.001	1.563 (0.926, 2.637)	0.094
Experiencing Covid Symptoms	1.670 (1.175, 2.373)	0.004	0.581 (0.332, 1.016)	0.057
Tested for Covid Before	1.890 (1.518, 2.354)	< 0.001	0.991 (0.626, 1.567)	0.968
Preferred Testing Method	0.751 (0.600, 0.941)	0.013	0.725 (0.463, 1.133)	0.158
Reporting Status	2.203 (1.767, 2.745)	< 0.001	1.553 (1.006, 2.396)	0.047

Outcome (Vaccination Status): fully vaccinated for Covid 19 vs. not fully vaccinated for Covid-19

Reference Categories for Covariates: Population Size – reference category was small population centre; Ethnicity - reference category was BIPOC; Income – reference category was > $75,000; Gender – reference category was nonbinary; Live with (# People) - reference category was > 5 people; Live with at Risk Persons – reference category was combination of conditions; Confirmed Contact with Covid – reference category was no confirmed contact; Experiencing Covid Symptoms – reference category was no symptoms; Tested for Covid before – reference category was not tested before; Preferred Testing Method – self test compared to reference category of clinical setting; Reported Covid Results - reference category was not reported

### Factors predicting COVID diagnosis

We evaluated predicting factors among individuals who reported a positive (*n* = 304) COVID self-test compared to those with a negative (*n* = 2441) result. We did not identify statistical significance by ethnicity among participants who had a positive or negative COVID self-test; indeed, the number of COVID diagnoses reported among white and BIPOC participants was fairly even: *n* = 152 positive tests from 2212 participants who identified as white (positivity rate of 6.9%), and *n* = 131 positive tests from 1905 participants who identified as BIPOC (positivity rate of 6.9%). Unsurprisingly, we identified that participants who reported a high-risk contact to COVID and who reported COVID-like symptoms were more likely to report a positive result. Geographic location was also found to be predictive, where participants from medium population centres (30,000–99,999 persons) were less likely to report a positive COVID self-test result compared to those from small population centres. (See Table 4).

**Table 4:** Results of Logistic Regression, with Binary Outcome Variable: Covid-19 Status

Covariates	Unadjusted		Adjusted	
	Odds Ratio (95% Confidence Interval)	*P*-Value	Odds Ratio (95% Confidence Interval)	*P*-Value
Population				
Large Urban	0.852 (0.604, 1.200)	0.359	0.701(0.489, 1.003)	0.052
Medium	0.403 (0.208, 0.782)	0.007	0.394 (0.202, 0.768)	0.006
Ethnicity	0.794 (0.620, 1.017)	0.068	0.786 (0.606, 1.019)	0.069
Age	0.989 (0.979, 0.999)	0.033	0.993 (0.982, 1.003)	0.161
Confirmed Contact with Covid	1.471 (1.128, 1.918)	0.004	1.374 (1.047, 1.804)	0.022
Experiencing Covid Symptoms	1.620 (1.201, 2.186)	0.002	1.507 (1.106, 2.054)	0.009

Outcome (Covid-19 Status): Predicting positive for Covid 19

Reference Categories for Covariates: Population Size – reference category was small population; Ethnicity - reference category was BIPOC; Confirmed Contact with Covid – reference category was no confirmed contact; Currently Experiencing Covid Symptoms – reference category was no symptoms

## Discussion

In September 2021, we launched GetaKit.ca/COVID [5] through which persons could complete an online risk assessment and obtain 1 or 2 free rt-LAMP COVID self-tests by mail, if eligible. Over the study period, 7781 orders were placed, of which 53% (*n* = 4160) were eligible; in total, we distributed 6469 COVID self-tests through these 4160 orders. These orders were relatively evenly distributed by ethnicity, with about half being from persons who identified as white and half being from participants who identified as BIPOC. Overall, 91% of participants reported full vaccination against COVID. Results were reported for 69% of the self-tests we had distributed, with 7.3% of these results being positive. Participants who reported higher incomes, prior COVID testing, full vaccination, and who lived with fewer than 5 people were more likely to have reported results back to GetaKit.ca/COVID. Participants who reported being a contact of someone diagnosed with COVID or who noted symptoms were, unsurprisingly, more likely to have reported a positive result. Lastly, only 56% of participants noted that self-testing was their preferred method for COVID testing. These results raise a few points for discussion.

First, our results shed some light on the potential limitations and utilities of online systems like GetaKit.ca/COVID to distribute screening tests for respiratory infections during a pandemic. On the one hand, undermining the success of this intervention is that GetaKit.ca/COVID primarily provided testing to persons who were previously connected to care. Over 90% of participants reported being fully vaccinated against COVID, and most had undergone prior testing for COVID by a healthcare provider. Despite outreach efforts and targeted awareness efforts for this pilot, very few test orders came from participants who had otherwise not previously been able to engage with the traditional healthcare system for COVID testing. While self-testing has been touted by some as a strategy to broaden access to testing, our results for mailout COVID testing did not bring in many first-time testers or persons without prior access to the healthcare system.

Moreover, most of the positive self-test results that were reported back to GetaKit.ca/COVID arose from participants either with COVID-like symptoms [8] or who were *bona fide* contacts [8] of someone diagnosed with COVID. In both cases, recommendations to self-isolate [1, 2, 8] could have yielded similar public health outcomes without requiring the use of testing resources. At the time we ran this study, local public health guidance [8] was that identification of negative self-test results did not mean people who had contact with someone diagnosed with COVID did not have to self-isolate or could end self-isolation earlier. In the absence of a change to public health practice or recommendations based on these results, the utility of self-testing for respiratory infections may be limited – and in fact do little more than consume resources.

On the other hand, our results suggest that such online or mobile health portals can also yield beneficial outcomes. Results supporting this include our uptake numbers, eligibility and positivity rates, and the fact that half of GetaKit.ca/COVID participants identified as BIPOC and 15% were from lower-income groups. This suggests that computer-mediated algorithms can be designed to correctly screen people in/out for testing [7, 11–13], including persons from the groups that were most affected by COVID infection and its sequalae [14–16]. While most of our orders for COVID self-tests arose from persons with prior engagement with the healthcare system, the algorithm did appropriately target testing, resulting in positivity rates that were comparable to those in COVID testing centres at the time [16]. That nearly half of our participants identified as BIPOC, when only 29% of persons in our jurisdiction (Ontario) are BIPOC [18], shows that systems such as GetaKit.ca/COVID can promote access for racialized persons. Rates of uptake among economically disadvantaged groups also indicates this service may have been able to facilitate access to healthcare for persons who, historically, have lower rates of engagement with this system [19, 20].

Furthermore, while online systems such as ours may not directly promote health equity by engaging with and providing services to all of the most marginalized members of our societies, these systems may inadvertently help equity-deserving populations by reducing demands from those who can use online services [13, 21]. That is, if people who are able to access online services opt to use them instead of seeking in-person care, websites like GetaKit.ca/COVID could free up time at in-person clinical and testing centres for individuals who require direct assistance (e.g., those without access to the Internet, those with disabilities that limit their use of self-testing or online systems, etc.). By providing access to high performance self-tests to people with Internet access and Internet literacy, clinicians would then not need to see them as patients. Clinicians could then provide assessments and testing for the aforementioned persons who are excluded by online systems and self-tests, such as anyone with visual impairments (who cannot read the self-test instructions or results) or persons with physical disabilities (that might limit their abilities to complete the manual steps required to perform the self-test). An important assumption underlying our supposition however is that in-person services must not be reduced due to online availability of testing [22]. If in-person care remains unchanged, then online services can route those who can use these services to them, while availing those who cannot access such services of more readily available in-person care. Clinicians can then provide direct services to those with the greatest needs for in-person care, while those who can and are willing, can obtain self-testing online.

Second, our results showed that, while uptake was high, half of our participants preferred to obtain COVID testing in traditional healthcare settings, and that this was not related to age, gender, ethnicity, or income. These findings highlight that self-testing is simply another option for testing that should be added to the array of testing strategies, and that it is not the main preference for testing for any distinct demographic group [22]. These findings also emphasize the need for decision-makers, healthcare professionals, and policy workers to continue to improve how healthcare services are provided so that everyone feels equally comfortable and welcome seeking services in these milieux [21, 22]. Promoting self-testing as the solution to discrimination within healthcare settings fails to address root cause issues [24, 25], including racism, sexism, homophobia, and transphobia, to name a few. We thus feel that our study to offer COVID self-tests highlighted that self-tests are an important option for some people – but not for all. Efforts are required to ameliorate healthcare, especially for those who experience the greatest barriers to accessing care and for those who experience the most negative experiences when receiving such care.

### Limitations

Our analysis of the use of an online system for distributing COVID-19 self test kits has a few limitations that warrant noting. First, this study occurred over a 7-month period in a single Canadian province and had a high concentration of participants from large population centres where COVID-19 testing locations may have been more accessible compared to medium or small population centres. It is possible that the outcomes we observed may have differed had the study run for longer or had recruitment or eligibility been targeted toward participants from rural or remote areas. Second, close to one-third of participants did not submit a self-test result, so it is the positivity rate for COVID infections could be higher than what is reported in these outcomes.

## Conclusion

In conclusion, our results identifed that, while self-testing should not be considered a panacea for infectious diseases, it can play an important role in healthcare service delivery if its limitations are acknowledged and addressed. For the limitations of self-testing, nearly half of our participants did not prefer this form of testing, and it was primarily only used by individuals who already had accessed the healthcare system for COVID testing. While self-testing is an important addition to the current testing armamentarium, policymakers and healthcare workers should not overly rely on this technology to remedy problems and shortcomings with current access. In other words, while self-testing may play a role, it cannot allow us to relax our efforts to improve healthcare systems by making them more accessible for everyone. The concern with relying too heavily on self-testing is that it could result in systems forcing marginalized groups to change how they obtain care (potentially by having them seek out inferior testing options), rather than forcing systems to become more inclusive based on race and ethnicity, sex and gender, and sexual orientation, to name a few. Despite these issues, our results show that online access to care can play a likely important role in modernizing how people obtain access to testing. Our project highlighted the potential of computer-algorithm driven systems like GetaKit.ca/COVID to facilitate appropriate and timely access to testing during respiratory pandemic situations – and to do so without requiring in-person access (thus reducing the risk of COVID transmission) and without further inundating beleaguered health human resources and clinical milieux. We take such results to mean that algorithm-driven risk assessments are a new tool to facilitate safe access to useable devices for self-screening during current conditions and during future pandemic situations. To maximize these benefits and minimize the limitations listed above, having these resources and systems tested and refined before another crisis is warranted. GetaKit.ca/COVID serves as one platform that can be built on as starting point for this work.

## Data Availability

The datasets used and/or analysed during the current study are available from the corresponding author on reasonable request.
